# Supplementary material for: Systematic review of predictive models of microbial water quality at freshwater recreational beaches
Source: PLoS One. 2021 Aug 26;16(8):e0256785. doi: 10.1371/journal.pone.0256785 (PMC8389397; doi:10.1371/journal.pone.0256785)
Supplement: S2 Table — (PDF) [file pone.0256785.s002.pdf]

S2 Table . Search terms used in each database.

Database	Search Terms
OVID/Medline	1. Water quality.mp or Water Microbiology or Water Quality<br>2. Escherichia coli.mp or Escherichia coli/<br>3. Enterobacteriaceae/ or Enterococcus/ or fecal indicator bacteria.mp or Feces/<br>4. 1 or 2 or 3<br>5. model, statistical.mp or Models, Statistical/<br>6. Forecasting/ or nowcast.mp<br>7. probability/ or regression analysis/ or regression.mp<br>8. regression.mp or Regression Analysis/<br>9. 5 or 6 or 7<br>9. Bathing Beaches/ or beach.mp<br>10. Environmental Monitoring/<br>11. weather/ or rain/ or temperature/ or weather.mp<br>12. 10 or 11<br>13. fresh water/ or lakes/ or ponds/ or rivers/<br>14. 9 or 10 or 13<br>15. 4 and 9 and 12 and 14
SciTech Premium	Noft(Escherichia coli OR enterococc* OR fecal indicator bacteria) AND noft(regression analysis OR predict* OR nowcast* OR forecast* OR model*) AND noft("fresh water" OR recreational water OR beach* OR lake OR river) AND noft(Weather OR monitor* OR rain* OR environmental)
Scopus	(Escherichia coli OR enterococc* OR fecal indicator bacteria) AND (regression analysis OR predict* OR nowcast* OR forecast* OR model*) AND ("fresh water" OR recreational water OR beach* OR lake OR river) AND (weather OR monitor* OR rain* OR environmental)
Web of Science	TS = ((Escherichia coli OR enterococc* OR fecal indicator bacteria) AND (regression analysis OR predict* OR nowcast* OR forecast* OR model*) AND ("fresh water" OR recreational water OR beach* OR lake OR river) AND (Weather OR monitor* OR rain* OR environmental))
ProQuest Dissertations and Thesis Global	Noft(Escherichia coli OR enterococc* OR fecal indicator bacteria) AND noft(regression analysis OR predict* OR nowcast* OR forecast* OR model*) AND noft("fresh water" OR recreational water OR beach* OR lake OR river) AND noft(Weather OR monitor* OR rain* OR environmental)
