# Supplementary material for: Systematic review of predictive models of microbial water quality at freshwater recreational beaches
Source: PLoS One. 2021 Aug 26;16(8):e0256785. doi: 10.1371/journal.pone.0256785 (PMC8389397; doi:10.1371/journal.pone.0256785)
Supplement: S3 Table — Searched December 10–14, 2020. (PDF) [file pone.0256785.s003.pdf]

S3 Table. **Grey literature search of government websites and their URLs. Searched December 10-14, 2020.**

**Organization**	**URL**
Government of Canada (search includes results from Health Canada, Public Health Canada, and Natural Resources Canada)	https://www.canada.ca/en/public-health.html
Center for Disease Control and Prevention	https://www.cdc.gov/nceh/ehs/publications/topic.htm
Ministry for the Environment, Government of New Zealand	https://www.mfe.govt.nz/fresh-water
US Geological Survey	https://www.usgs.gov/
US Environmental Protection Agency	https://www.epa.gov/
Public Health England	https://www.gov.uk/
Public Health Europe	https://ec.europa.eu/health/home_en<br>https://swimproject.eu/reports/
Scotland's Environment Web	https://www.environment.gov.scot/
World Health Organization	https://apps.who.int/iris/
