# Supplementary material for: Systematic review of predictive models of microbial water quality at freshwater recreational beaches
Source: PLoS One. 2021 Aug 26;16(8):e0256785. doi: 10.1371/journal.pone.0256785 (PMC8389397; doi:10.1371/journal.pone.0256785)
Supplement: S4 Table — (PDF) [file pone.0256785.s004.pdf]

S4 Table . Eligibility criteria to define microbes of interest, geography, predictors of interest, and types of publications.

Inclusion	Exclusion
Fecal indicator bacteria (ex. E.coli, enterococcus, any other gastrointestinal microorganisms used to predict the presence of pathogens)	Modeling of non-pathogenic species (zooplankton, fish, etc.), chemical toxicants, cyanobacteria and associated toxins
Fresh water, inland beaches	Marine, coastal, or estuarial beaches. Drinking water quality. Natural waters not used for recreational swimming. Treated or chlorinated waters.
Temperate climatic region	Tropics, subtropics, artic climatic regions
Statistical models based on weather, environment, or climatic data (rainfall, windspeed, solar radiation, temperature, wave height)<br>Model is used to predict risk for use by bathers and/or beach managers	Models based only on previous day FIB levels, genetic tests (qPCR), or other culture techniques
Report, primary article, conference proceeding, thesis, or dissertation reporting on primary research	Commentary, any other work not reporting on primary research
