# Supplementary material for: Systematic review of predictive models of microbial water quality at freshwater recreational beaches
Source: PLoS One. 2021 Aug 26;16(8):e0256785. doi: 10.1371/journal.pone.0256785 (PMC8389397; doi:10.1371/journal.pone.0256785)
Supplement: S5 Table — (PDF) [file pone.0256785.s005.pdf]

S5 Table . Data extraction form, including primary outcomes and risk of bias questions

Section	Question	Answers
Study Characteristics	What year was the article published?	
	Where were the beaches located? (Region/city, province/state, country)	
	Number of beaches included in analysis	
	Number of swimming seasons' data used in model building	
Analytical Methods	Type of statistical model(s) used in final analysis	○ Multilinear regression<br>○ Bayesian networks<br>○ Artificial neural networks<br>○ Tree regression and/or random forests<br>○ Other: _____
	Possible explanatory variables assessed	○ Rainfall <24 hr<br>○ Rainfall 24hr<br>○ Rainfall 48 hr<br>○ Rainfall 72+ hr<br>○ Temperature<br>○ Wave height<br>○ River/stream outflow of water<br>○ Sewer outflow [FIB]<br>○ Previous day [FIB]<br>○ Solar radiation<br>○ Barometric pressure<br>○ Turbidity<br>○ Wind speed<br>○ Wind direction<br>○ Relative humidity<br>○ Discharge/flow (m3/s)<br>○ Conductivity<br>○ pH<br>○ Chlorophyll a<br>○ Total nitrogen<br>○ Total phosphorus<br>○ Other:_______
	Explanatory variables used in final model	
	Laboratory testing used, type of bacterial indicator, and how the bacterial concentration is reported	○ E. coli<br>○ Enterococcus<br>○ Other:______<br><br>○ Categorical<br>○ Continuous<br>○ Log transformed
	Performance measurements	Sensitivity =<br>Specificity =<br>R^2 =

		AUC (c-statistic) =<br>Other performance measures: __
	Has this model been used in practice?	○ Yes, with data publicly available<br>○ Yes, with data kept internally<br>○ No<br>○ Unsure
	What was the method of measuring predictors/ source of predictors?	○ Governmental data<br>○ Collected by beach management<br>○ Conservation Authorities<br>○ Measured by researchers<br>○ Unsure
	Type of predictors in model	○ Continuous<br>○ Categorical<br>○ Transformed<br>If transformed, reason for transforming: ______
	Handling of missing data	Handling of missing data: ___
	Are the modelling assumptions satisfied?	○ Yes<br>○ No<br>○ Unsure
	Were predictor weights adjusted to address overfitting of model?	○ Yes<br>○ No<br>○ Not applicable<br>○ Unsure
	How was the model validated?	○ Bootstrapping<br>○ Division of original dataset<br>○ Temporal validation<br>○ Geographical validation
	Were the distributions of predictors compared for development and validation datasets?	○ Yes<br>○ No<br>○ Unsure
	Predictor selection method (including preselection methods if relevant)	○ Full model approach<br>○ Backward elimination<br>○ Forward selection<br>○ Akaike Information Criterion<br>○ Bayesian Information Criterion<br>○ C-index<br>○ Nominal p-value<br>○ Other: ________
