# Supplementary material for: Systematic review of predictive models of microbial water quality at freshwater recreational beaches
Source: PLoS One. 2021 Aug 26;16(8):e0256785. doi: 10.1371/journal.pone.0256785 (PMC8389397; doi:10.1371/journal.pone.0256785)
Supplement: S6 Table — (PDF) [file pone.0256785.s006.pdf]

S6 Table. Descriptive summary of number of swimming seasons used for model building, number of beaches investigated, FIB of interest, geography of beaches, and type of publication of the 53 relevant studies.

Study characteristics	Number of studies	% of studies
Number of swimming seasons used in building model		
1	19	36%
2	7	13%
3	8	15%
4	7	13%
5	1	2%
6	4	8%
7	3	6%
8 or more	3	6%
Unspecified	1	2%
Number of beaches / sampling sites investigated		
1	17	32%
2	5	9%
3	10	19%
4	6	11%
5	3	6%
6-10	4	8%
10-20	4	8%
20-50	3	6%
Unspecified	1	2%
Fecal indicator bacteria of interest		
E. coli	46	87%
Enterococcus	11	21%
Fecal coliforms	1	2%
Other bacteria	1	2%
Country of beach(es)		
U.S.	44	83%
Germany	4	8%
Canada	2	4%
New Zealand	2	4%
France	1	2%
Body of water modelled		
Lake	40	75%
River	13	25%
Recreational waters investigated		
Lake Michigan	20	38%
Lake Erie	14	26%
Inland Ohio lakes	5	9%
Other rivers in U.S.	5	9%
Cuyahoga River, U.S.	4	8%
Ruhr River, Germany	3	6%
Other lakes in U.S.	3	6%

Lake Superior	2	4%
Lake Ontario	2	4%
Other German rivers	2	4%
Great Lakes, unspecified	1	2%
Lakes in France	1	2%
Lakes in New Zealand	1	2%
Rivers in New Zealand	1	2%
Other lakes in Canada	1	2%
Type of publication		
Journal article	34	64%
Government report	12	23%
Thesis	3	6%
Dissertation	2	4%
Conference proceeding	2	4%
