# Supplementary material for: Systematic review of predictive models of microbial water quality at freshwater recreational beaches
Source: PLoS One. 2021 Aug 26;16(8):e0256785. doi: 10.1371/journal.pone.0256785 (PMC8389397; doi:10.1371/journal.pone.0256785)
Supplement: S7 Table — (PDF) [file pone.0256785.s007.pdf]

S7 Table. Frequency of variables explored in studies and used in a final model for predicting microbial water quality.

Variable	Number of studies that explored the variable (n)	% of total studies	Number of times used in at least one final model of a study (n)	% of studies variable was explored in that included it in a final model
**Weather**				
Rainfall	45	85%	39	87%
Rainfall 24hr	35	66%	20	57%
Rainfall 48 hr	25	47%	17	68%
Rainfall 72+ hr	18	34%	15	83%
Rainfall <24 hr	16	30%	8	50%
Number of days since last rainfall/ number of dry days	10	19%	7	70%
Rainfall intensity	3	6%	2	67%
Wind direction	28	53%	25	89%
Wind speed	28	53%	23	82%
Air temperature	25	47%	12	48%
Cloud cover	13	25%	9	69%
Solar radiation	22	42%	9	41%
Barometric pressure	10	19%	7	70%
Relative humidity	7	13%	4	57%
Weather category	3	6%	2	67%
Dew point	4	8%	1	25%
Sunny category	1	2%	1	100%
**Water conditions**				
Turbidity	36	68%	31	86%
Water temperature	37	70%	18	49%
Algae index/ accumulation/ presence	7	13%	5	71%
Debris category	3	6%	2	67%
Carlson's trophic index	1	2%	1	100%
Odor	1	2%	1	100%
Secchi depth	1	2%	1	100%

**Hydrodynamics**				
Wave height	27	51%	24	89%
Water level	23	43%	12	52%
Current direction	16	30%	8	50%
Current speed	11	21%	4	36%
Wave direction	5	9%	3	60%
Wave period/ frequency	7	13%	2	29%
**Contamination sources**				
Stream discharge/ flow (m^3/s)	29	55%	18	62%
Bird count	16	30%	12	75%
Bather count	8	15%	7	88%
Previous day FIB	7	13%	4	57%
Fecal matter	2	4%	2	100%
Boat count	2	4%	1	50%
Sewer overflow	2	4%	1	50%
Streamflow FIB	1	2%	1	100%
**Temporal variables**				
Day of year/ Julian day	13	25%	13	100%
Sampling time of day	4	8%	4	100%
Sub-season/ month	4	8%	3	75%
Day of week	1	2%	1	100%
**Water chemistry**				
Conductivity	19	36%	6	32%
pH	14	26%	5	36%
Chlorophyll *a*	7	13%	3	43%
Dissolved oxygen	11	21%	2	18%
Ammonium ion	2	4%	2	100%
Nitrate	3	6%	2	67%
Phosphorus or phosphate	3	6%	2	67%
Spectral absorption	2	4%	1	50%
**No variables listed**	2	4%	4	200%
