# Supplementary material for: Systematic review of predictive models of microbial water quality at freshwater recreational beaches
Source: PLoS One. 2021 Aug 26;16(8):e0256785. doi: 10.1371/journal.pone.0256785 (PMC8389397; doi:10.1371/journal.pone.0256785)
Supplement: S8 Table — (PDF) [file pone.0256785.s008.pdf]

S8 Table. Average accuracy of models that assessed accuracy and whether or not they performed better than persistence models

Authors and year of publication	Average accuracy (%)	Performance compared to persistence model(s)
Brady, Amie M G, Bushon, Rebecca N, Plona, Meg B (2009)	77.5	Modelling at sites performed better than persistence but expanding models to new locations performed worse
Brady, Amie M G, Plona, Meg B (2009)	77.5	Unknown
Brady, Amie M.G., Plona, Meg B. (2015)	78.6	All 8 models better than persistence models
Dada, Ayokunle Christopher, Hamilton, David P (2016)	96.67	No persistence models reported, and very few exceedances in validation set
Francy, Donna S., Darner, Robert A., Bertke, Erin E. (2006)	82.5	All but one model outperformed persistence
Francy, Amie M. Gifford, and Robert A. Darner (2003)	81.1	All but one model outperformed persistence
Francy, Donna S, Stelzer, Erin A, Duris, Joseph W, Brady, Amie M G, Harrison, John H, Johnson, Heather E, Ware, Michael W (2013)	78.1	3 of 9 models statistically better than persistence (1 had zero exceedances)
Francy, Donna S., Bertke, Erin E., Darner, Robert A. (2006)	73	Both models outperformed persistence
He, Cheng, Post, Yvonne, Dony, John, Edge, Tom, Patel, Mahesh, Rochfort, Quintin (2016)	77.4	All 5 years of models better than current method
Jones, Rachael M, Liu, Li, Dorevitch, Samuel (2013)	75.5	Unknown
Madani, M, Seth, R (2020)	72.6	All 10 models outperformed persistence models
Maimone, Mark, Crockett, Christopher S, Cesanek, William E (2007)	66.0	Unknown
Nevers, Meredith B, Whitman, Richard L (2005)	97.5	Better than persistence
Olyphant, G A (2005)	86.8	All four models outperformed persistence
Shively, Dawn A, Nevers, Meredith B, Breitenbach, Cathy, Phanikumar, Mantha S, Przybyla-Kelly, Kasia, Spoljaric, Ashley M, Whitman, Richard L (2016)	83.4	10 of 14 models had higher accuracy than persistence
Simmer, Reid A (2016)	87.3	Both models outperformed persistence
Uejio, Christopher K, Peters, Theodore W, Patz, Jonathan A (2012)	84.1	Unknown
US Geological Survey (2003)	82.9	Unknown
Wang, Leizhi, Zhu, Zhenduo, Sassoubre, Lauren, Yu, Guan, Liao, Chen, Hu, Qingfang, Wang, Yintang (2020)	80.4	Unknown
