# Supplementary material for: Systematic review of predictive models of microbial water quality at freshwater recreational beaches
Source: PLoS One. 2021 Aug 26;16(8):e0256785. doi: 10.1371/journal.pone.0256785 (PMC8389397; doi:10.1371/journal.pone.0256785)
Supplement: S9 Table — (PDF) [file pone.0256785.s009.pdf]

S9 Table . Risk-of-bias characteristics of 53 articles reporting on predictive models of fecal indicator bacteria using environmental predictors, excluding characteristics found in Table 1 of main text.

Author and year of publication	Source of predictor data	Method for selecting predictors for multivariate modeling	Predictor selection method for inclusion in final model	Were predictor weights or regression coefficients shrunk at all?	Are modeling assumptions satisfied?	Was the outcome assessed with blinded explanatory variables?	Were predictors blinded for outcome and each other?	Handling of predictors in modelling (transformations)	Handling of missing data	Were predictor distributions compared between calibration and validation datasets?
Anderson, Kendall W (2019)	Governmental data, Measured by researchers	All included	full model approach	NA	Unsure	No	No	12hr rain fall into wet and dry periods, Categorized	Days with missing predictor data removed from analysis (3 day)	No
Avila, Rodelyn, Horn, Beverley, Moriarty, Elaine, Hodson, Roger, Moltchanova, Elena (2018)	Unsure	Unknown	Unknown	NA	Unsure	No	No	NA	Left as missing	No

Bachmann-Machnik, Anna, Dittmer, Ulrich, Schoenfeld, Annika (2019)	Unsure	Univariate model	Univariate model	NA	Unsure	No	No	Na	NA	No
Brady, Amie M G, Bushon, Rebecca N, Plona, Meg B (2009)	Governmental data, Measured by researchers	All included	Model fit characteristics	NA	Unsure	No	No	Log10 turbidity	NA	No
Brady, Amie M G, Plona, Meg B (2009)	Governmental data, Measured by researchers	Univariate model	Univariate model	NA	Unsure	No	No	Log10 turbidity	NA	No
Brady, Amie M.G., Plona, Meg B. (2015)	Conservation Authorities, Measured by researchers	Virtual Beach	Virtual Beach	NA	Unsure	No	No	Log10 turbidity, square root of 48hr rain in first model	NA	No
Brady, Amie MG, Plona, Meg B (2012)	Governmental data, Conservation Authorities	All included	Model fit characteristics	NA	Yes	No	No	Log10 turbidity, first 24hr of rain in 48hr weighted more than second	NA	No

Brooks, Wesley R., Fienen, Michael N., Corsi, Steven R. (2013)	Governmental data, Collected by other researchers in past studies	All included	Full model approach	NA	Unsure	No	No	Log10 turbidity, Categorized	Left as missing	No
Brooks, Wesley, Corsi, Steven, Fienen, Michael, Carvin, Rebecca (2016)	Governmental data, Measured by researchers	All included	Adaptive LASSO, genetic algorithm, and AIC	NA	Unsure	No	No	Log10 turbidity, log10 river discharge, and square root of rainfall based on previous studies, Categorized	Drop variables with missing data	No
Corsi, Steven R, Borchardt, Mark A, Carvin, Rebecca B, Burch, Tucker R, Spencer, Susan K, Lutz, Michelle A, McDermott, Colleen M, Busse, Kimberly M, Kleinheinz,	Governmental data, Measured by researchers	Least absolute shrinkage and selection operator	None	NA	Unsure	No	No	Categorized	Variables with missing data removed from analysis	No

Gregory T, Feng, Xiaoping, Zhu, Jun (2016)										
Cyterski, M, Zhang, S, White, E, Molina, M, Wolfe, K, Parmar, R, Zepp, R (2012)	Governmental data, Measured by researchers, Other researchers in past studies	Sets of highly correlated predictors filtered	AIC and other model fit characteristics	NA	Unsure	No	No	NA	NA	No
Dada, Ayokunle Christopher, Hamilton, David P (2016)	Governmental data, Other researchers in past studies	Virtual Beach	Virtual Beach	NA	Unsure	No	No	Weight adjusted rainfall, square distance from lake, square root 96hr rainfall, square suspended solids, Categorized	NA	No
Donna S. Francy, Amie M. Gifford, and Robert A. Darner (2003)	Governmental data, Other researchers in past studies	All included	Model fit characteristics	NA	Unsure	No	No	Rainfall and weighted rainfall included, log10 turbidity, sine and cosine of wind direction, Categorized	Left as missing	No

Francy, D.S., Brady, A.M.G., Carvin, R.B., Corsi, S.R., Fuller, L.M., Harrison, J.H., Hayhurst, B.A., Lant, J., Nevers, M.B., Terrio, P.J., Zimmerman, T.M. (2013)	Governmental data	Virtual Beach	Virtual Beach	NA	Unsure	No	No	Categorized	Missing weather data replaced with data from next nearest weather station	No
Francy, Donna S, Darner, Robert A (2007)	Governmental data, Measured by researchers	Unknown	Unknown	NA	Unsure	No	No	Weighted rainfall, log10 turbidity	Left as missing	No
Francy, Donna S, Stelzer, Erin A, Duris, Joseph W, Brady, Amie M G, Harrison, John H, Johnson, Heather E, Ware,	Governmental data, Measured by researchers	Virtual Beach	Virtual Beach	NA	Unsure	No	No	NA	NA	No

Michael W (2013)										
Francy, Donna S., Bertke, Erin E., Darner, Robert A. (2009)	Governmental data, Measured by researchers	All included	Model fit characteristics	NA	Unsure	No	No	Rain weighted 48hr = (2*Rain day 1) + (1*Rain day 2), log10 turbidity	Left as missing	No
Francy, Donna S., Darner, Robert A., Bertke, Erin E. (2006)	Governmental data, Measured by researchers	Only univariately significant variables included	Model fit characteristics	NA	Unsure	No	No	Weighted rainfall, log10 turbidity	NA	No
Frick, W.E (2006)	Unsure	Virtual Beach	Virtual Beach	NA	Unsure	No	No	NA	NA	No
Frick, Walter E., Ge, Zhongfu, Zepp, Richard G. (2008)	Governmental data	Virtual Beach	Virtual Beach	NA	Unsure	No	No	Categorized	NA	No
Hatfield, Nancy Lee Clark (2000)	Governmental data	All included	Stepwise, unspecified	NA	Unsure	No	No	Rainfall category: 1=sunny calm day, 2=drizzle, 3=rain, and 4=thunderstorm	NA	No

He, Cheng, Post, Yvonne, Dony, John, Edge, Tom, Patel, Mahesh, Rochfort, Quintin (2016)	Governmental data	Only univariately significant variables included	Forward selection	NA	Yes	No	No	Categorized	NA	No
Heberger, Matthew G, Durant, John L, Oriel, Kimberly A, Kirshen, Paul H, Minardi, Lee (2008)	Governmental data	All included	Stepwise, unspecified	NA	Unsure	No	No	log10 (Precipitation+ 1 inch), log10 (time since last rainfall)	NA	No
Herrig, Ilona, Seis, Wolfgang, Fischer, Helmut, Regnery, Julia, Manz, Werner, Reifferscheid, Georg, Boeer, Simone (2019)	Governmental data, Measured by researchers	All included	Model fit characteristics	NA	Yes	No	No	NA	Missing sensor values from instrument failure replaced with in situ values collected during FIB sample selection	Yes

Hong, Yi, Soulignac, Frederic, Roguet, Adelaide, Li, Chenlu, Lemaire, Bruno J, Martins, Rodolfo Scarati, Lucas, Francoise, Vincon-Leite, Brigitte (2021)	Measured by researchers	All included	Full model approach	NA	Unsure	No	No	NA	NA	No
Jones, Rachael M, Liu, Li, Dorevitch, Samuel (2013)	Other researchers in past studies	All included	Tree regression and random forests with conditional inference trees	NA	Unsure	No	No	Rain variables log10 transformed to fit AIC better	NA	No
Madani, M, Seth, R (2020)	Governmental data	Preselected variables based on literature	model fit characteristics and AIC	NA	Unsure	No	No	RainCombine = β1*Rain8hr + β2*Rain12hr + β3*Rain24hr + β4*Rain48hr + β5*Rain72hr, Categorized	NA	No

Maimone, Mark, Crockett, Christopher S, Cesanek, William E (2007)	Governmental data, Measured by researchers, Other researcher in past studies	All included	Examining trends in data	NA	Unsure	No	No	NA	NA	No
Mälzer, H.-J., aus der Beek, T, Müller, S, Gebhardt, J (2016)	Water supplier and wastewater treatment plant	Unsure	Full model approach for ANN, unsure for others	NA	Unsure	No	No	NA	NA	No
Marion, Jason W (2011)	Measured by researchers	Univariate model	Univariate model	NA	Yes	No	No	NA	NA	No
Molina, M.,, Cyterski, Mike, Whelan, G., Zepp, R. (2014)	Governmental data, Measured by researchers	Unknown	Unknown	NA	Unsure	No	No	NA	NA	No
Motamarri, Srinivas, Boccelli, Dominic L (2012)	Other researcher in past studies	All included	Backwards elimination	NA	Unsure	No	No	Log10 discharge	NA	No

Nevers, Meredith B, Shively, Dawn A, Kleinheinz, Gregory T, McDermott, Colleen M, Schuster, William, Chomeau, Vinni, Whitman, Richard L (2009)	Governmental data	All included	Model fit characteristics	NA	Yes	No	No	Categorized	NA	No
Nevers, Meredith B, Whitman, Richard L (2005)	Governmental data, Measured by researchers	All included	Stepwise, unspecified	NA	Unsure	No	No	NA	NA	No
Nevers, Meredith B, Whitman, Richard L, Frick, Walter E, Ge, Zhongfu (2007)	Governmental data, Measured by researchers	Removed redundant variables and ones without enough days	AIC	NA	Unsure	No	No	Categorized	NA	No
Nevers, Meredith B., Whitman,	Governmental data, Measured	Preselected based on independence and	AIC	NA	Unsure	No	No	Z-score of average turbidity and	NA	No

Richard L. (2008)	by researchers	colinearity with other variables						wave height, Categorized		
Olyphant, G A (2005)	Measured by researchers	All included	Model fit characteristics	NA	Yes	No	No	Categorized	NA	No
Olyphant, Greg A, Whitman, Richard L (2004)	Measured by researchers	Only univariately significant variables included	Full model approach	NA	No	No	No	NA	NA	No
Parkhurst, David F, Brenner, Kristen P, Dufour, Alfred P, Wymer, Larry J (2005)	Unsure	All included	Full model approach	NA	Yes	No	No	Categorized	Left as missing	No
Rossi, Alessandra, Wolde, Bernabas T., Lee, Lee H., Wu, Meiyin (2020)	Governmental data	All included	Forward selection	NA	Unsure	No	No	NA	NA	No
Safaie, Ammar, Wendzel, Aaron, Ge,	Governmental data, Measured	All included	Both full model (mechanistic) and	NA	Yes	No	No	ln(turbidity) and	NA	No

Zhongfu, Nevers, Meredith B, Whitman, Richard L, Corsi, Steven R, Phanikumar, Mantha S (2016)	by researchers		backward elimination (statistical)					ln(turbidity of discharge)		
Seis, W, Zamzow, M, Caradot, N, Rouault, P (2018)	Governmental data	Chosen based on *a priori* criteria	Most models unsure, one model forward selection	NA	Yes	No	No	Log10 rain, variable for average values 1,2,3,4, and 5 days back for each variable made	NA	Yes
Shively, Dawn A, Nevers, Meredith B, Breitenbach, Cathy, Phanikumar, Mantha S, Przybyla-Kelly, Kasia, Spoljaric, Ashley M, Whitman, Richard L (2016)	Measured by researchers	All included	AIC, BIC, and other model fit characteristics, with a maximum number of variables set to 5	NA	Unsure	No	No	Categorized	Missing solar data replaced with nearby sensor, buoy and turbidimeter data left as missing, resulted in missing *E. coli* predictions at 2 beaches during one season preventing accurate	No

									validation there	
Simmer, Reid A (2016)	Measured by researchers, Other researcher in past studies	Virtual Beach	Virtual Beach	NA	Yes	No	No	Rain weighted 48hr = (2*Rain day 1) + (1*Rain day 2), day of year squared, water temperature polynomial, dissolved O2 polynomial, weighted 72hr rainfall polynomial, alongshore wind quadroot, onshore wave polynomial, pH polynomial, beachgoers squared, goose droppings quadroot, humidity polynomial	NA	No
Telech, Justin W, Brenner, Kristen P, Haugland, Rich, Sams, Elizabeth,	Governmental data, Measured by researchers	All included	Backwards elimination	NA	Unsure	No	No	Categorized	NA	No

Dufour, Alfred P, Wymer, Larry, Wade, Timothy J (2009)										
Uejio, Christopher K, Peters, Theodore W, Patz, Jonathan A (2012)	Governmen tal data	All included	Backward Stepwise Deviance Information Criterion	NA	Yes	No	No	Categorized	Autocorrelati on and partial autocorrelati on	Yes
US Geological Survey (2003)	Governmen tal data	Only univariately significant variables included	Model fit characteristi cs	NA	Unsure	No	No	sum of the sines of wind direction, weighted rainfall	Missing *E. coli* data removed previous day FIB from variable list	No
Wang, Leizhi, Zhu, Zhenduo, Sassoubre, Lauren, Yu, Guan, Liao, Chen, Hu, Qingfang, Wang, Yintang (2020)	Governmen tal data	Both unknown and all included based on model	Both unknown and all included based on model	NA	Unsure	No	No	Rainfall weighted (in addition to unweighted), Categorized	Missing *E. coli* left as missing	No

Wendzel, Aaron (2014)	Measured by researchers	All included	Full model approach	NA	Yes	No	No	NA	NA	No
Whitman, R L, Nevers, M B (2008)	Governmental data, Conservation Authorities	All included	AIC	NA	Unsure	No	No	NA	Left as missing	No
Zhang, Juan, Qiu, Han, Li, Xiaoyu, Niu, Jie, Neyers, Meredith B, Hu, Xiaonong, Phanikumar, Mantha S (2018)	Unsure	All included	Full model approach	NA	Unsure	No	No	Log10 turbidity, log10 river discharge	NA	No
Zimmerman, Tammy M (2008)	Governmental data, Measured by researchers	Only univariately significant variables included	Model fit characteristics	NA	Unsure	No	No	Log10 turbidity	NA	No
Zimmerman, Tammy M (2006)	Governmental data, Measured by researchers	Only univariately significant variables included	Backward and forward selection	NA	Unsure	No	No	Weighted rainfall, log turbidity, Categorized	NA	No
