# Supplementary material for: Systematic review of predictive models of microbial water quality at freshwater recreational beaches
Source: PLoS One. 2021 Aug 26;16(8):e0256785. doi: 10.1371/journal.pone.0256785 (PMC8389397; doi:10.1371/journal.pone.0256785)
Supplement: S1 Protocol — (PDF) [file pone.0256785.s010.pdf]

Protocol for literature review

Predictive Models of Water Quality at Freshwater Recreational Beaches for Management Decisions: Protocol for a Systematic Review

Cole Heasley[1], J. Johanna Sanchez[1,] Jordan Tustin[1], Ian Young[1]

[1]Ryerson University, School of Occupational and Public Health

Financial support: Funding for the project has been provided by the Public Health Agency of Canada. The funder had no role in this review.

Rationale

Between 2000 and 2014, 140 outbreaks at untreated recreational water sources were reported in the United States, leading to 4958 cases, with 84% of the outbreaks associated with a lake/pond/reservoir (Graciaa et al., 2018). Fecal indicator bacteria (FIB), often *Escherichia coli,* are sampled at recreational beaches as a marker of potential pathogen concentrations and risk of infection to bathers. However, the traditional method of culturing bacteria takes 18-24 hours to reach a result, meaning beach managers are making water quality decisions using previous day measurements. More modern genetic techniques, such as qPCR, can achieve results in 3-4 hours but are expensive for public health units to run daily (Shrestha & Dorevitch, 2020). Some beaches have moved to predicting FIB levels using predictive models. Recent examples of predictive modelling approaches use a popular method developed by the US Geological Survey, called nowcasting, uses environmental inputs such as temperature, precipitation, and turbidity to predict *E. coli* levels at beaches (Francy et al., 2013).

While there are many pathogens that are spread via recreational water use, such as *Campylobacter, Salmonella,* norovirus, and *Cryptosporidium*, *E.coli* is often used in models as indicator of the presence of these other pathogens (Health Canada, 2012). *Enterococcus* is sometimes used for predictions in addition to or in place of *E. coli* (Government of Australia National Health and Research Council, 2008; Jones, Liu, & Dorevitch, 2013; World Health Organization, 2003). However, *E.* coil is often used as an indicator in freshwater due to a strong correlation between *E. coli* concentrations and increased risk of gastrointestinal illness in swimmers (Health Canada, 2012; Marion, Lee, Lemeshow, & Buckley, 2010).

A wide variety of predictive modelling methods have been used in recreational beaches; including multiple linear regression (Madani & Seth, 2020; Shively et al., 2016), artificial neural networks (Zhang et al., 2018), and Bayesian networks (Mellios, Moe, & Laspidou, 2020). These models use local weather data that are correlated with fecal indicator bacteria concentrations in the water (Jones et al., 2013; Nevers & Whitman, 2011). Predictor variables have included rainfall in the past 24-48 hours, temperature, solar radiation, sewage and river outflows, and wave height at the beach, among others. While some research has investigated which of several models is best for a particular beach (Avila, Horn, Moriarty, Hodson, & Moltchanova, 2018), there is a lack of studies looking into models across beaches. The purpose of this review is to identify what modeling methods used, where they have been applied, and how they compare in their sensitivity and specificity to correctly predicting beach management decision guidelines (leaving beach open or closing it due to water quality issues).

Objectives

To present a summary of predictive models using environmental variables used to predict fecal indicator bacteria concentrations at recreational freshwater beaches.

Research Questions

1. What types of predictive models were created for predicting water quality and fecal indicator bacteria concentrations based on environmental variables for beach management decisions?

2. Which predictors or covariates were included in these models?

3. How accurate are the models for determining if recreational beach water exceeds water quality guidelines compared to traditional culture or genetic-based methods?

Methods

This review will follow the PRISMA-P 2015 checklist (Shamseer et al., 2015).

Search Strategy

Table S2. Search terms used in each database.

Database	Search Terms
OVID/Medline	1. Water quality.mp or Water Microbiology or Water Quality<br>2. Escherichia coli.mp or Escherichia coli/<br>3. Enterobacteriaceae/ or Enterococcus/ or fecal indicator bacteria.mp or Feces/<br>4. 1 or 2 or 3<br>5. model, statistical.mp or Models, Statistical/<br>6. Forecasting/ or nowcast.mp<br>7. probability/ or regression analysis/ or regression.mp<br>8. regression.mp or Regression Analysis/<br>9. 5 or 6 or 7<br>9. Bathing Beaches/ or beach.mp<br>10. Environmental Monitoring/<br>11. weather/ or rain/ or temperature/ or weather.mp<br>12. 10 or 11<br>13. fresh water/ or lakes/ or ponds/ or rivers/

	14. 9 or 10 or 13<br>15. 4 and 9 and 12 and 14
SciTech Premium	Noft(Escherichia coli OR enterococc* OR fecal indicator bacteria) AND noft(regression analysis OR predict* OR nowcast* OR forecast* OR model*) AND noft("fresh water" OR recreational water OR beach* OR lake OR river) AND noft(Weather OR monitor* OR rain* OR environmental)
Scopus	(Escherichia coli OR enterococc* OR fecal indicator bacteria) AND (regression analysis OR predict* OR nowcast* OR forecast* OR model*) AND ("fresh water" OR recreational water OR beach* OR lake OR river) AND (weather OR monitor* OR rain* OR environmental)
Web of Science	TS = ((Escherichia coli OR enterococc* OR fecal indicator bacteria) AND (regression analysis OR predict* OR nowcast* OR forecast* OR model*) AND ("fresh water" OR recreational water OR beach* OR lake OR river) AND (Weather OR monitor* OR rain* OR environmental))
ProQuest Dissertations and Thesis Global	Noft(Escherichia coli OR enterococc* OR fecal indicator bacteria) AND noft(regression analysis OR predict* OR nowcast* OR forecast* OR model*) AND noft("fresh water" OR recreational water OR beach* OR lake OR river) AND noft(Weather OR monitor* OR rain* OR environmental)

Table S3. Grey literature search of government websites and their URLs. Searched December 10-14, 2020.

**Organization**	**URL**
Government of Canada (search includes results from Health Canada, Public Health Canada, and Natural Resources Canada)	https://www.canada.ca/en/public-health.html
Center for Disease Control and Prevention	https://www.cdc.gov/nceh/ehs/publications/topic.htm
Ministry for the Environment, Government of New Zealand	https://www.mfe.govt.nz/fresh-water
US Geological Survey	https://www.usgs.gov/
US Environmental Protection Agency	https://www.epa.gov/
Public Health England	https://www.gov.uk/
Public Health Europe	https://ec.europa.eu/health/home_en<br>https://swimproject.eu/reports/
Scotland's Environment Web	https://www.environment.gov.scot/
World Health Organization	https://apps.who.int/iris/

Handsearching:

References of articles selected for the review will be hand searched.

Table S4. Eligibility criteria to define microbes of interest, geography, predictors of interest, and types of publications.

Inclusion	Exclusion
Fecal indicator bacteria (ex. E.coli, enterococcus, any other gastrointestinal microorganisms used to predict the presence of pathogens)	Modeling of non-pathogenic species (zooplankton, fish, etc.), chemical toxicants, cyanobacteria and associated toxins
Fresh water, inland beaches	Marine, coastal, or estuarial beaches. Drinking water quality. Natural waters not used for recreational swimming. Treated or chlorinated waters.
Temperate climatic region	Tropics, subtropics, artic climatic regions
Statistical models based on weather, environment, or climatic data (rainfall, windspeed, solar radiation, temperature, wave height)<br>Model is used to predict risk for use by bathers and/or beach managers	Models based only on previous day FIB levels, genetic tests (qPCR), or other culture techniques
Report, primary article, conference proceeding, thesis, or dissertation reporting on primary research	Commentary, any other work not reporting on primary research

Study Selection

Data management: citations and abstracts will be uploaded from databases into Mendeley. Screening of titles and abstracts, and full articles will be done in DistillerSR.

Round 1: Two reviewers screen titles and abstracts

- Is this article relevant to the review question? [Yes/No/Unsure]

Round 2: Two reviewers screen full articles

- Is this article about microbial water quality? (Measuring E. coli, enterococcus, cyanobacteria, other water-borne illnesses) [Yes/No]
- Is this article about freshwater recreational beaches? [Yes/No]
- Does this article report on a predictive model for beach water quality using environmental data? [Yes/No]

Data Characterization and Extraction

Data will be obtained by CH into a form in DistillerSR involving the data extraction elements (below).

Primary Outcomes:

The type of predictive model (e.g. regression analysis, Bayesian network), the variables inputted into the model, and the performance metrics used to evaluate the model.

Risk of Bias

Risk of bias will be assessed using the **CH**ecklist for critical **A**ppraisal and data extraction for systematic **R**eviews of prediction **M**odelling **S**tudies (CHARMS) (Moons et al., 2014). The checklist will be adapted from human health predictive models to environmental modeling.

Additional information extraction included in appendix.

Data synthesis/ summary

A descriptive and tabular summary of the methods, variables, and performances of predictive models will be presented. While we will report on performance metrics we will not draw conclusions on validity beyond the risk of bias.

Table S5. Data extraction form, including primary outcomes and risk of bias questions

Section	Question	Answers
Study Characteristics	What year was the article published?	
	Where were the beaches located? (Region/city, province/state, country)	
	Number of beaches included in analysis	
	Number of swimming seasons' data used in model building	
Analytical Methods	Type of statistical model(s) used in final analysis	○ Multilinear regression<br>○ Bayesian networks<br>○ Artificial neural networks<br>○ Tree regression and/or random forests<br>○ Other: ____
	Possible explanatory variables assessed	○ Rainfall <24 hr<br>○ Rainfall 24hr<br>○ Rainfall 48 hr<br>○ Rainfall 72+ hr<br>○ Temperature<br>○ Wave height<br>○ River/stream outflow of water<br>○ Sewer outflow [FIB]<br>○ Previous day [FIB]<br>○ Solar radiation<br>○ Barometric pressure<br>○ Turbidity<br>○ Wind speed<br>○ Wind direction<br>○ Relative humidity<br>○ Discharge/flow (m3/s)<br>○ Conductivity
	Explanatory variables used in final model	

		○ pH<br>○ Chlorophyll a<br>○ Total nitrogen<br>○ Total phosphorus<br>○ Other:______
	Laboratory testing used, type of bacterial indicator, and how the bacterial concentration is reported	○ E. coli<br>○ Enterococcus<br>○ Other:_____<br><br>○ Categorical<br>○ Continuous<br>○ Log transformed
	Performance measurements	Sensitivity =<br>Specificity =<br>R^2 =<br>AUC (c-statistic) =<br>Other performance measures: __
	Has this model been used in practice?	○ Yes, with data publicly available<br>○ Yes, with data kept internally<br>○ No<br>○ Unsure
	What was the method of measuring predictors/ source of predictors?	○ Governmental data<br>○ Collected by beach management<br>○ Conservation Authorities<br>○ Measured by researchers<br>○ Unsure
	Type of predictors in model	○ Continuous<br>○ Categorical<br>○ Transformed<br>If transformed, reason for transforming: ______
	Handling of missing data	Handling of missing data: ___
	Are the modelling assumptions satisfied?	○ Yes<br>○ No<br>○ Unsure
	Were predictor weights adjusted to address overfitting of model?	○ Yes<br>○ No<br>○ Not applicable<br>○ Unsure
	How was the model validated?	○ Bootstrapping<br>○ Division of original dataset<br>○ Temporal validation<br>○ Geographical validation
	Were the distributions of predictors compared for development and validation datasets?	○ Yes<br>○ No<br>○ Unsure

	Predictor selection method (including preselection methods if relevant)	○ Full model approach<br>○ Backward elimination<br>○ Forward selection<br>○ Akaike Information Criterion<br>○ Bayesian Information Criterion<br>○ C-index<br>○ Nominal p-value<br>○ Other: _______

Bibliography

Avila, R., Horn, B., Moriarty, E., Hodson, R., & Moltchanova, E. (2018). Evaluating statistical model performance in water quality prediction. *Journal of Environmental Management*, *206*, 910–919. https://doi.org/10.1016/j.jenvman.2017.11.049

Francy, D. S., Brady, A. M. G., Carvin, R. B., Corsi, S. R., Fuller, L. M., Harrison, J. H., … Zimmerman, T. M. (2013). *Developing and implementing predictive models for estimating recreational water quality at Great Lakes beaches. U.S. Geological Survey Scientific Investigations Report 2013-5166*. Retrieved from https://pubs.usgs.gov/sir/2013/5166/

Government of Australia National Health and Research Council. (2008). *Guidelines for Managing Risks in Recreational Water*.

Graciaa, D. S., Cope, J. R., Roberts, V. A., Cikesh, B. L., Kahler, A. M., Vigar, M., … Hlavsa, M. C. (2018). Outbreaks associated with untreated recreational water — United States, 2000–2014. *MMWR. Morbidity and Mortality Weekly Report*, *67*(25), 701–706. https://doi.org/10.15585/mmwr.mm6725a1

Health Canada. (2012). Guidelines for Canadian Recreational Water Quality – Third Edition- Part II: Guideline Technical Documentation. Retrieved November 25, 2020, from https://www.canada.ca/en/health-canada/services/publications/healthy-living/guidelines-canadian-recreational-water-quality-third-edition/guidelines-canadian-recreational-water-quality-third-edition-page-9.html#part2

Jones, R. M., Liu, L., & Dorevitch, S. (2013). Hydrometeorological variables predict fecal indicator bacteria densities in freshwater: Data-driven methods for variable selection. *Environmental Monitoring and Assessment*, *185*, 2355–2366. https://doi.org/10.1007/s10661-012-2716-8

Madani, M., & Seth, R. (2020). Evaluating multiple predictive models for beach management at a freshwater beach in the Great Lakes region. *Journal of Environmental Quality*, *49*, 896–908. https://doi.org/10.1002/jeq2.20107

Marion, J. W., Lee, J., Lemeshow, S., & Buckley, T. J. (2010). Association of gastrointestinal illness and recreational water exposure at an inland U.S. beach. *Water Research*, *44*(16), 4796–4804. https://doi.org/10.1016/j.watres.2010.07.065

Mellios, N. K., Moe, S. J., & Laspidou, C. (2020). Using Bayesian hierarchical modelling to capture cyanobacteria dynamics in Northern European lakes. *Water Research*, *186*, 116356. https://doi.org/10.1016/j.watres.2020.116356

Moons, K. G. M., de Groot, J. A. H., Bouwmeester, W., Vergouwe, Y., Mallett, S., Altman, D. G., … Collins, G. S. (2014). Critical Appraisal and Data Extraction for Systematic Reviews of Prediction Modelling Studies: The CHARMS Checklist. *PLoS Medicine*, *11*(10). https://doi.org/10.1371/journal.pmed.1001744

Nevers, M. B., & Whitman, R. L. (2011). Efficacy of monitoring and empirical predictive modeling at improving public health protection at Chicago beaches. *Water Research*, *45*(4), 1659–1668. https://doi.org/10.1016/j.watres.2010.12.010

Shamseer, L., Moher, D., Clarke, M., Ghersi, D., Liberati, A., Petticrew, M., … Whitlock, E. (2015). Preferred reporting items for systematic review and meta-analysis protocols (prisma-p) 2015: Elaboration and explanation. *BMJ*, *349*(g7647). https://doi.org/10.1136/bmj.g7647

Shively, D. A., Nevers, M. B., Breitenbach, C., Phanikumar, M. S., Przybyla-Kelly, K., Spoljaric, A. M., & Whitman, R. L. (2016). Prototypic automated continuous recreational water quality monitoring of nine Chicago beaches. *Journal of Environmental Management*, *166*, 285–293. https://doi.org/10.1016/j.jenvman.2015.10.011

Shrestha, A., & Dorevitch, S. (2020). Slow adoption of rapid testing: Beach monitoring and notification using qPCR. *Journal of Microbiological Methods*, *174*, 105947. https://doi.org/10.1016/j.mimet.2020.105947

World Health Organization. (2003). *Guidelines for safe recreational water environments VOLUME 1 COASTAL AND FRESH WATERS The World Health Organization's (WHO) new Guidelines for Safe Recreational Water*. Retrieved from https://apps.who.int/iris/bitstream/handle/10665/42591/9241545801.pdf?sequence=1

Zhang, J., Qiu, H., Li, X., Niu, J., Nevers, M. B., Hu, X., & Phanikumar, M. S. (2018). Real-Time Nowcasting of Microbiological Water Quality at Recreational Beaches: A Wavelet and Artificial Neural Network-Based Hybrid Modeling Approach. *Environmental Science and Technology*, *52*(15), 8446–8455. https://doi.org/10.1021/acs.est.8b01022
