# Supplementary material for: Systematic review of predictive models of microbial water quality at freshwater recreational beaches
Source: PLoS One. 2021 Aug 26;16(8):e0256785. doi: 10.1371/journal.pone.0256785 (PMC8389397; doi:10.1371/journal.pone.0256785)
Supplement: Attachment — Submitted filename: REVIEW NOTE-PONE-D-21-14797.docx [file pone.0256785.s011.docx]

Interactive comment on “***Systematic Review of Predictive Models of Microbial Water Quality at Freshwater Recreational Beaches*” by Heasley et al.**

**Pr. Salim Heddam**

[*heddamsalim@yahoo.fr*](https://mail.google.com/mail/u/0/h/10407zvxwxrzf/?&cs=wh&v=b&to=heddamsalim@yahoo.fr)*.*

[https://orcid.org/0000-0002-8055-8463](https://www.google.com/url?q=https%3A%2F%2Forcid.org%2F0000-0002-8055-8463&sa=D&sntz=1&usg=AFQjCNGUSPuWiGbgHOPCWiCtmwUB7RsjnQ)

The paper is very interesting, well written and well documented and easy to read. Few similar studies are available in the literature. In depth literature review was conducted and a useful taxonomy of predictive models of microbial water quality at freshwater (i.e. lakes and river) was provided, where standards regression and machines learning models are categorized into several groups based upon their: structure, input variables (i.e., predictor), location, number of beaches and swimming seasons, performances metrics and how the proposed models were validated against measured data, and especially, highlighting their practical benefit and environmental implications. Also, the present study provides a taxonomy that can be used to distinguish between simple, complicated, and complex models developed at different time scale. I have seen some important conclusion of the present study, which I really appreciate: (*i*) multiple linear regressions is used most often at nearly 70%, (*ii*) rainfall is reported as the most important weather variable used for model development, (*iii*) water turbidity (68%) and temperature (70%) are the most significant water quality variables selected as relevant predictors, (*iv*) in overall the proposed models were validated using R^2^ and the RMSE, while what surprised me mostly is that, the Nash-Sutcliffe efficiency was rarely adopted as performance metric for model validation, and (*v*) the most fecal indicator of interest is the *E.coli.* The reviewed papers were deeply analyzed and the reported results were scientifically discussed which made the manuscript very sound.

I have read the paper several times, and I see that the authors have hardly worked for providing an excellent paper and to my scientific opinion it can be accepted without revision.
