# Supplementary material for: Systematic review of predictive models of microbial water quality at freshwater recreational beaches
Source: PLoS One. 2021 Aug 26;16(8):e0256785. doi: 10.1371/journal.pone.0256785 (PMC8389397; doi:10.1371/journal.pone.0256785)
Supplement: Attachment — Submitted filename: Response to Reviewers.docx [file pone.0256785.s012.docx]

August 4, 2021

PloS ONE (PONE-D-21-14797)

Dear Dr Zaher Mundher Yaseen and Reviewers,

We would like to thank you for your time in reviewing our manuscript and for your feedback. We have provided responses to your comments in italics and blue, in addition to updates to the manuscript. Please note line references in the is letter correspond to the tracked document.

Best,

Cole Heasley

Corresponding author

Reponses to editor:

1. Please ensure that your manuscript meets PLOS ONE's style requirements, including those for file naming. The PLOS ONE style templates can be found at

<https://journals.plos.org/plosone/s/file?id=wjVg/PLOSOne_formatting_sample_main_body.pdf> and

<https://journals.plos.org/plosone/s/file?id=ba62/PLOSOne_formatting_sample_title_authors_affiliations.pdf>

*Article title was unbolded and only the first word is now capitalized.*

*Affiliations updated to match style guide and corresponding author added.*

*Heading and subheading font increased as per style guide. Figure and table titles bolded, with their intext citations unbolded.*

2. Please include captions for your Supporting Information files at the end of your manuscript, and update any in-text citations to match accordingly. Please see our Supporting Information guidelines for more information: http://journals.plos.org/plosone/s/supporting-information.

*Tables in supplementary information in separate PDFs. A file for the whole protocol is still included as its own PDF file as well. Supporting information caption added to end of review. In text citations of supporting information updated to match PLoS ONE style guide.*

Reviewer #1:

The paper is very interesting, well written and well documented and easy to read. Few similar studies are available in the literature. In depth literature review was conducted and a useful taxonomy of predictive models of microbial water quality at freshwater (i.e. lakes and river) was provided, where standards regression and machines learning models are categorized into several groups based upon their: structure, input variables (i.e., predictor), location, number of beaches and swimming seasons, performances metrics and how the proposed models were validated against measured data, and especially, highlighting their practical benefit and environmental implications. Also, the present study provides a taxonomy that can be used to distinguish between simple, complicated, and complex models developed at different time scale. I have seen some important conclusion of the present study, which I really appreciate: (i) multiple linear regressions is used most often at nearly 70%, (ii) rainfall is reported as the most important weather variable used for model development, (iii) water turbidity (68%) and temperature (70%) are the most significant water quality variables selected as relevant predictors, (iv) in overall the proposed models were validated using R2 and the RMSE, while what surprised me mostly is that, the Nash-Sutcliffe efficiency was rarely adopted as performance metric for model validation, and (v) the most fecal indicator of interest is the E.coli. The reviewed papers were deeply analyzed and the reported results were scientifically discussed which made the manuscript very sound.

I have read the paper several times, and I see that the authors have hardly worked for providing an excellent paper and to my scientific opinion it can be accepted without revision.

*NA*

Reviewer #2: The topic of this review (Systematic Review of Predictive Models of Microbial Water Quality at Freshwater Recreational Beaches) is interesting and informative for the readers. This manuscript has been written reasonably and satisfactorily, but further modifications are required before publication. My comments are:

- The abstract needs improvements and add more mathematical findings to be more informative.

*While also attempting to stay within the word count limits, we included a summary of key results, including numerical data on specific outcomes, to highlight the most relevant findings in the abstract. If the reviewer has more specific suggestions of what to highlight, we would be happy to consider those.*

- A list of abbreviations should be added.

*A list of abbreviations has been added between the conflicts of interest and references section.*

- A list of contents should be included.

*We believe a list of contents is added automatically by the journal when it is published.*

-The novelty of this work was not clearly presented. Please follow the literature review and show the knowledge gaps identified and link them to your research objectives.

*Further comments have been added to clarify the novelty of the review in lines 235 and 243.*

- The conclusion part needs improvement to make it more informative to the readership.

*Several sentences were added to the conclusion to make it more informative.*

Reviewer #3:

Abstract:
Comment No.1: Don’t use He, we in all the manuscript, revise the following sentences. And other sentences in all the manuscript. And so on.
*Upon review of other systematic reviews, including those published in PLoS One, we noted the common use of third person pronouns. As other papers in PLoS ONE use “we” and it provides an easier use of the active voice our preference is to also use this.*

Comment No.2: Add recommendation in the nd of the abstract for future research.

*Another recommendation on creation of a non-medical risk of bias checklist has been added to the end of the abstract.*

Introduction:
Comment No.3: Check that all references cited on the manuscript.

*We have checked and all references are cited in the manuscript.*

Results:
Comment No.4: Add column in the left of Table 1 indicates the number of study from 1 to 53

*A numbered column has been added to the table.*

Comment No.5: Add column in the right in Table 1 shows the recommendations if found of each study

*A column has been added to Table 1 with the conclusions/ recommendations of each study from their abstracts and/or conclusions.*

Comment No.6: Add column in the center of Table 1 displays the limitations of using the proposed model of each study if found

*Limitations of individual studies are presented in the risk of bias checklist (Table S9 in the supplementary information).*

Comment No.7: Use pie chart and column charts beside with figures to visualize your results in Table 2, 3, and 4
Especially for Table No.4, for each section, new figure can be added
Weather, hydrodynamic, contamination sources and others

*Several bar charts have been added detailing some of these tables (figs 2 – 6). Not all of the data in tables were included, such as table 3 (now table 2), as we believe not much information would be gained with a visualization due to how skewed the modelling techniques are towards linear regression. We have also used bar charts instead of pie charts as they are the preferred chart type for displaying categorical data.*

*Tables containing the same data as presented in figures have been moved to the supplementary information.*

Comment No.8: Use pie chart to indicate results for each contour.

*We are unsure what the reviewer is referring to by “contour”.*

Comment No.9: In your question: Were predictor weights or regression coefficients shrunk at all?
Indicate why the results 100% for No answer

*Thank you for the comment, an error was caught as one study did use this practice. More information was added to specify this practice refers to regularization of data. Otherwise, this was a step that was simply not used or not mentioned in the articles.*

Comment No.10: Add new section and talk about the accuracy and limitations of predictive models.

*The accuracy of models is discussed in the paragraph after figure 6, line 205. Limitations of the predictive models, based on amount of data, poor validation choices, and their performance are in table 1 and in table S9 in the supplementary information. There were no overarching limitations mentioned in the articles other than what was already included.*

Comment No.11: For results in table number 5, indicate the limitation with researchers comparing with governmental for the availability of data and its effect on results.

*We assume the reviewer is looking for an explanation on if using governmental data rather than data collected on the beach by researchers has any effect on model results. A sentence has been added to the discussion in line 273 discussing that this is likely not an issue as most models are likely sensitive enough to have major differences from small changes to parameters that one would expect from sampling at different locations.*

Comment No.12: Add new table ion it compare between the used models and each limitations in prediction.

*We are unsure that adding this table would add any new information to the manuscript since it would repeat information already presented in two other tables.*

Discussion:
Provide reasons for the following conclusions:
Comment No.13: Multiple linear regression methods were the most popular and were shown to produce accurate predictions. However, other methods may produce more accurate predictions. Comparing models built at different locations with different variables and rates of FIB exceedances would not yield accurate comparisons; however, four studies included in this review compared modelling techniques.

*Additional detail was added to the last sentence to clarify that the conclusions are explained in the following two sentences.*

Comment No.14: Add sentences indicate the less significant of other parameters when using the modeling rather than the parameters in the following sentences:
Larger waves may also be responsible for washing bird fecal matter from the beach into the water [54]. Wind direction and speed are important explanatory variables as they are associated with driving FIB from sediments or point sources towards the beach [77,78]. Winds, waves, and turbidity are often correlated 277 parameters, as winds and waves churn sediments which increases turbidity [43,78].

*We are unsure what the reviewer is asking for in this comment and request further clarification.*

Comment No.15: Give more information about the following sentence:
Different geographical contexts require different approaches and variables, so it is important to explore these elements in new contexts.

*This paragraph was rearranged to better explain this concluding sentence, as well more information was added to it to improve clarity.*

Conclusion:
Comment No.16: Add one more recommendations for future studies in the end of conclusion section.

*Another recommendation for creating a risk of bias checklist specific for non-medical predictive models.*

Comment No.17: Add symbol section definition if available to you.

*A list of abbreviations was added after the conflicts of interest declaration, however no symbols were used in the manuscript.*
